# SiNPle: Fast and Sensitive Variant Calling for Deep Sequencing Data

**DOI:** 10.3390/genes10080561

**Published:** 2019-07-25

**Authors:** Luca Ferretti, Chandana Tennakoon, Adrian Silesian, Graham Freimanis, Paolo Ribeca

**Affiliations:** Integrative Biology and Bioinformatics, The Pirbright Institute, Woking GU24 0NF, UK

**Keywords:** next generation sequencing, low-frequency variants, heterogeneous populations, Bayesian modelling

## Abstract

Current high-throughput sequencing technologies can generate sequence data and provide information on the genetic composition of samples at very high coverage. Deep sequencing approaches enable the detection of rare variants in heterogeneous samples, such as viral quasi-species, but also have the undesired effect of amplifying sequencing errors and artefacts. Distinguishing real variants from such noise is not straightforward. Variant callers that can handle pooled samples can be in trouble at extremely high read depths, while at lower depths sensitivity is often sacrificed to specificity. In this paper, we propose SiNPle (Simplified Inference of Novel Polymorphisms from Large coveragE), a fast and effective software for variant calling. SiNPle is based on a simplified Bayesian approach to compute the posterior probability that a variant is not generated by sequencing errors or PCR artefacts. The Bayesian model takes into consideration individual base qualities as well as their distribution, the baseline error rates during both the sequencing and the PCR stage, the prior distribution of variant frequencies and their strandedness. Our approach leads to an approximate but extremely fast computation of posterior probabilities even for very high coverage data, since the expression for the posterior distribution is a simple analytical formula in terms of summary statistics for the variants appearing at each site in the genome. These statistics can be used to filter out putative SNPs and indels according to the required level of sensitivity. We tested SiNPle on several simulated and real-life viral datasets to show that it is faster and more sensitive than existing methods. The source code for SiNPle is freely available to download and compile, or as a Conda/Bioconda package.

## 1. Introduction

Detection of low-frequency variants is an important area in the downstream analysis of high-throughput sequencing. In cancer studies, it can provide means of detecting circulating cancer cells and be helpful in the early diagnosis and prognosis, or to detect relapse. It is also useful for the study of DNA populations, for example to analyse cancer heterogeneity and the evolution of viral quasi-species [1]. Study of genetic variation in heterogeneous samples is another research area that has been facilitated by recent technological advances, making it possible to generate high coverage data that enable deep sequencing and detection of low-frequency variants. For example, a routine sequencing run of a small RNA virus such as foot-and-mouth disease virus can return about 5000-fold coverage of its genome. In addition, in scenarios involving targeted sequencing, for example when performing targeted resequencing of liquid biopsy samples [2], an extremely high coverage of the targeted regions are common. However, sample preparation protocols and sequencing methods are error prone. Illumina sequencing implies a base calling error rate of up to 1–2%. Various artefacts may also be generated during sample preparation and get amplified during PCR stages. These artefactual variants can be significantly represented in the sequencing data as coverage becomes very high. Furthermore, the nature and rates of these errors differ between sequencing platforms, sample preparation protocols, sequencing runs and sometimes even between the lanes of the same run. Therefore, naive search for low frequency variants without taking into account the sources of errors often results in calling a large number of non-existent variants.

Some kinds of sequencing artefacts can be removed using computational tools for adapter trimming [3] and (depending on the nature of the sample) by removing duplicate reads [4]. It is also a relatively easy problem to call heterozygous variants in diploid samples or fixed differences with respect to the reference genome, as they should have an expected allele frequency of 50% or 100% and there are many well-established solutions available to do so [5]. However, the underlying assumptions for the low-frequency variant calling problem are very different and require a different statistical approach.

The general method to call SNPs and short indels is to generate a stack of read alignments—a pileup—for each position in the genome. This pileup can be inspected for variations from the reference genome. Statistical modelling is then used to evaluate whether the observed variation is real or an artefact. The statistical model often takes into account simple features of the variant such as base quality, frequency or allele count.

There are several established ways of calling variants. Methods such as VarScan2, VarDict and SOAPsnv [6,7,8,9,10] first locate putative variants by checking if reads covering them satisfy certain criteria (for example, a minimum number of reads supporting the variant). These criteria are set to rule out variants arising due to expected errors while sequencing. At this point, statistical tests and/or heuristic rules are applied to identify high confidence variants.

Other variant callers such as LoFreq and deepSNV [11,12] deal with variant calling as a hypothesis testing problem. The null hypothesis assumes that the bases are from reference or a consensus. The alternative hypothesis is that the bases are from a variant. The observed count of non-reference reads is taken as the test statistic. LoFreq treats each base as the result of independent Bernoulli trials with success being a non-reference base and the probability determined by the quality score. Callers such as deepSNV and LoLoPicker [13] are good at calling variants in targeted sequencing data. They do not rely on quality scores to infer error rates, and assume that the error rate is either a random variable or fixed for a given site.

In this paper, we propose SiNPle (Simplified Inference of Novel Polymorphisms from Large coveragE), a simple, fast and effective approach to discriminate true variants from artefacts based on approximate Bayesian modelling. The model takes into account several factors: (i) individual base qualities and their distribution; (ii) baseline error rates (both during sequencing and during PCR); (iii) prior distribution of variant frequencies; and (iv) strandedness. SiNPle outputs summary statistics for each variants, including the approximate posterior probabilities from the Bayesian model. We tested our code on simulated and real deep sequencing data from viral samples, showing that SiNPle has the advantage of being very fast even for high coverage data, and compares favourably in terms of specificity and sensitivity with existing variant callers such as VarScan2 and LoFreq.

## 2. Materials and Methods

### 2.1. SiNPle Algorithm

For the sake of simplicity, we employ an approximate Bayesian approach based on multiple biallelisations of alleles at each position. For samples at high ploidy sequenced at high read depth, such as viruses, most sites are multiallelic. However, common alleles are easily detected as true variants, while the likelihoods of rare alleles are approximately independent on each other. More precisely, in a Bayesian context, the posterior probability of true and false variants at a given site can be approximated by the product of marginal probabilities up to a factor $1+\sum_{i}O\left(f_{i}\right)$ where $f_{i}$ are the frequencies of the minor variants. Hence, we can assess the probability that a given allele represents a true variant in the population by considering each allele independently at a given site. In practice, for each variant, we collapse all the reads not containing the variant into a single “non-variant” classification and we compare the frequency and quality of reads containing the variant with this “non-variant” set—hence projecting the content of the site into a biallelic space—and repeating the process for each variant at the site. We denote the read depth by *r*, the count of the variant *v* by $c_{v}$, its frequency by $f_{v}=c_{v}/r$ and its average quality by $q_{v}$.

Our Bayesian approach consider two models for each allele at each site: either the variant is actually present in the sample (“true variant”) or it is generated by amplification or sequencing errors (“error”). The computation for the posterior probability P[truevariant] is based on the Bayesian formula:

(1) $$P\left[\mathrm{true}\;\mathrm{variant}\right]=\frac{P\left[\mathrm{true}\;\mathrm{variant}|\mathrm{pileup}\right]P_{0}\left[\mathrm{true}\;\mathrm{variant}\right]}{P\left[\mathrm{true}\;\mathrm{variant}|\mathrm{pileup}\right]P_{0}\left[\mathrm{true}\;\mathrm{variant}\right]+P\left[\mathrm{error}|\mathrm{pileup}\right]P_{0}\left[\mathrm{error}\right]}$$

where the pileup represents the set of data about the content of all reads covering a given position, including counts of each allele and their qualities.

The priors are defined in terms of the prior probability θ that a random variant is actually present in the sampled population. In detail, we define the prior probability of a real variant as $P_{0}\left[\mathrm{true}\;\mathrm{variant}\right]=\theta$, while the complementary probability is $P_{0}\left[\mathrm{error}\right]=1-\theta$. Note that, for neutral populations, this parameter is different from the population genetics parameter with the same name and it is approximately given by $2N_{e}\mu \left(\gamma +\log(\min\left(\bar{r},n\right))\right)$ where $N_{e}$ is the effective (haploid) population size, μ is the mutation rate per base, γ is the Euler–Mascheroni constant ≈0.58, *n* is the number of individuals in the sample and $\bar{r}$ is the average read depth.

The likelihood of a real variant P[truevariant|pileup] can be decomposed into the likelihoods of the read count for the variant, multiplied by the likelihood $L_{Q}\left[\mathrm{pileup}\right]$ that the distribution of qualities is consistent with the distribution for correctly sequenced bases. According to classical population genetics, the frequency distribution of derived alleles is proportional to the inverse of the frequency [14], hence the population genetic equation for the likelihood of finding a polymorphic site of derived allele count $c_{d}$ in a population of *r* neutrally evolving individuals is ${\left(c_{d}\sum_{c=1}^{r}1/c\right)}^{-1}$ [15]. Since $c_{v}=\min\left(c_{d},r-c_{d}\right)$, the likelihood has the form:

(2) $$P\left[\mathrm{true}\;\mathrm{variant}|\mathrm{pileup}\right]=L_{Q}\left[\mathrm{pileup}\right]\frac{r}{c_{v}\left(r-c_{v}\right)\sum_{c=1}^{r}1/c}$$

To compute $L_{Q}\left[\mathrm{pileup}\right]$, we assume that all quality distributions are Gaussian. This is a strong simplification compared to the actual distribution of qualities, but it has the advantage of being independent on the shape of the distribution. First, we compute the mean and variance of the null distribution from the corresponding bases in the reads in the “non-variant” class. Then, we remove quality values in the lower 25% of the distribution for the variant, in order to remove sequencing errors (that tend to represent a larger fraction of bases even in real low-frequency variants). If the mean of this truncated distribution is higher than the null one, we define it to be equal to the null one in order to avoid penalisation of high-quality variants. Finally, we compute $L_{Q}\left[\mathrm{pileup}\right]$ as the Gaussian likelihood for the mean of the truncated distribution. Note that, for indels, since a distribution of base qualities is not available, we define $L_{Q}\left[\mathrm{pileup}\right]=1$.

The likelihood of different types of errors P[error|pileup] can be decomposed into two contributions.

1. The likelihood that each base of the variant arises as a sequencing error: There are two possible approaches to estimate it, starting from the baseline error rate ϵ and under the approximate assumption that all “non-variant” alleles are correct:
  (3) P[error|pileup]seq.errors,1=rcvϵcv(1−ϵ)r−cv≈rcvϵcve−ϵ(r−cv)
  or based on the base qualities (or equivalent qualities for indels) of the variant:
  (4) $$P{\left[\mathrm{error}|\mathrm{pileup}\right]}_{\mathrm{seq}.\mathrm{errors},2}=10^{-c_{v}q_{v}/10}$$
  To obtain a more conservative estimate, we sum the likelihoods from both approaches.
2. The likelihood that the variant is actually a PCR artefact generated by a mutation during the PCR: The density of the frequency distribution of mutations arising from a binary exponential PCR amplification process is approximately $2\epsilon_{\mathrm{PCR}}/f_{v}^{2}$ where $\epsilon_{\mathrm{PCR}}$ is the mutation rate per base during the PCR process. After discretising the distribution over *r* reads, the likelihood becomes
  (5) $$P{\left[\mathrm{error}|\mathrm{pileup}\right]}_{\mathrm{PCR}}=L_{Q}\left[\mathrm{pileup}\right]\frac{2\epsilon_{\mathrm{PCR}}r}{c_{v}^{2}}$$

The likelihoods are then combined as $P\left[\mathrm{error}|\mathrm{pileup}\right]=P{\left[\mathrm{error}|\mathrm{pileup}\right]}_{\mathrm{seq}.\mathrm{errors}}+P{\left[\mathrm{error}|\mathrm{pileup}\right]}_{\mathrm{PCR}}$.

Indels are classified as short (single base insertion or deletion) or long (insertion or deletion of multiple bases). These two sets have different parameters for baseline error rate $\epsilon_{\mathrm{indel},\mathrm{short}}$ and $\epsilon_{\mathrm{indel},\mathrm{long}}$ but a single PCR error rate $\epsilon_{\mathrm{PCR},\mathrm{indel}}$ and a single prior for the probability of true indels $\theta_{\mathrm{indel}}$. Moreover, while qualities are assigned as miscalling probabilities to individual bases within indels, no quality is assigned by the machine to the actual presence of the insertion/deletion. Hence, we assume for simplicity that they have fixed qualities described by the parameters $q_{\mathrm{indel},\mathrm{short}}$ and $q_{\mathrm{indel},\mathrm{long}}$.

If the sequencing is directional, we use only reads that map to the appropriate strand.

SiNPle was implemented in the OCaml programming language. Its source code is freely available for download at [16], and a packaged version is also available on Conda/BioConda.

### 2.2. Programs Setup

SiNPle was run setting the prior for sequencing error θ to the values 0.001 (default) and another, more sensitive value (0.01 or 0.1), depending on the dataset. It should be noted that setting θ to 0 discards all variants while θ=1 accepts all of them, thus, if the value for θ used in the sensitive mode were large enough, this procedure would be guaranteed to span virtually all the ROC space and generate an essentially complete ROC curve. For LoFreq and VarScan2, SNP predictions were generated setting the *p*-value thresholds at 0.01,0.02,…,0.99 and at 0.001,0.0001 and 0.00001, respectively. In addition to their conservative default settings, VarScan2 and LoFreq were also run tuning their parameters to make them more sensitive. For VarScan2, the strand filtering was switched off, the minimum coverage and the quality to call a variant was set to 1 and the minimum frequency of the variant was lowered to 0.0001. For LoFreq, all the post filtering of variants was switched off.

### 2.3. Assessing Specificity and Sensitivity of Variant Calling on Simulated Data

We used simulated data to assess the predictions of the variant callers versus a known ground truth. While the best way to design a realistic simulation is not universally agreed upon, we used existing simulators to generate viral populations and sample NGS reads from them. With the FTEC [17] program, we generated three poliovirus populations using an exponential growth model, each containing 5000 viruses and having mutation rates of 0.6%, 1% and 5%, respectively. We then generated 400,000 virus copies for each dataset using a PCR duplication simulator written in R. The final set of reads was simulated with ART [18], and each PCR-processed copy of the viruses was used as the reference for the reads produced from it. We generated Illumina reads of length 200 bp and random reads were collected into datasets with coverages 100, 500, 1000 and 5000. The end result is a set of 12 datasets having coverages 100, 500, 1000 and 5000 sampled from populations with SNP rates 0.6%, 1% and 5%. The ART simulator already generates the exact alignment of each reads. We used these alignments and their pileups as input to the variant callers. We have made these datasets available on our website.

LoFreq and VarScan2 were run on their default mode and in their sensitive modes as described in the previous section. The sensitive mode for SiNPle was obtained by setting θ=0.1.

### 2.4. Assessing Specificity and Sensitivity of Variant Calling on Real Data

In general, assessing the specificity and sensitivity of low-frequency variant callers is not an easy task for real data, as the number of predictions can be very large and experimentally validating them with low-throughput methods would be challenging. Several methods have been used in the past, for instance highly parallel PCR systems [11].

CirSeq is a sequencing protocol that can be used to detect low-frequency variants in RNA viruses [19]. It circularises fragmented viral RNAs and performs rolling-circle RT on these fragments. The result is a read containing tandem repeats of the same fragment, typically three copies per read. By examining the consensus of the three tandem repeats and their qualities, CirSeq can predict the actual sequence and assign a confidence to each base with much more precision than one would be able to do just by looking at the pileup of the three copies without linkage. That happens because variants appearing repeatedly in the same read at the same position on more than one of the copies of the fragment are much more likely to be real variants than sequencing errors. Thus, CirSeq represents an ideal experimental technique when evaluating low-frequency variant callers.

For our validation, we used the CirSeq poliovirus dataset available on the NCBI SRA [20] under accession number SRR1036990. It contains about 53 million reads of length 323. From this dataset, we randomly sampled 100,000 reads; that amounts to a coverage of ∼4000 of the poliovirus, which is representative of typical sequencing scenarios. The tandem repeats of RNA fragments within each read were identified by aligning the read to the reference with BLAST, and converted to a standard SAM file, thus breaking linkage between the repeats and obtaining reads of ∼100 bases each.

The pileup and BAM file for this SAM file was then used as the input for all variant callers.

**Figure 1:**
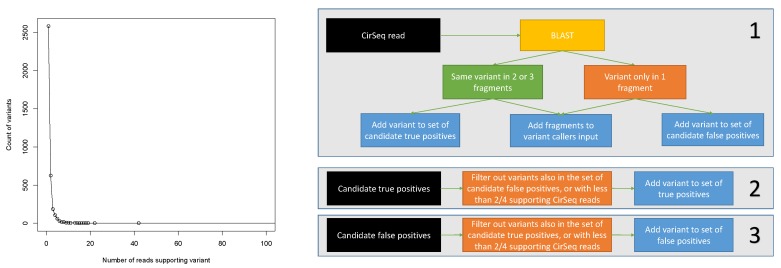
(**Left**) Number of variants as a function of the number of CirSeq reads supporting them. (**Right**) Schematic depiction of the procedure used to determine the sets of true and false positive variants from CirSeq data.

To reliably call variants from CirSeq data using linkage information, we used the following method depicted in Figure 1. We started from the reads aligned to the poliovirus genome by BLAST for which variants with respect to the reference sequence could be identified in the alignment of at least one fragment. If all the three fragments aligned to the reference without indels, we computed the consensus sequence for the fragments. If some variant was present in the consensus, it meant that the variant was present in at least two of the three segments, and we then added it to the list of candidate true positives—given the very low overall probability of sequencing error in Illumina sequencing, such a variant is much more likely to be real rather than a sequencing error. On the other hand, if a variant was only present in one of three segments (and thus not appearing in the consensus), it was a likely sequencing error, and we added it to the list of candidate false positives. We also kept track of the number of reads supporting each variant for both lists. After processing all CirSeq reads, we subtracted from both lists the intersection of the lists—i.e., we subtracted the variants being both a candidate true positive and a candidate false negative, as their classification is questionable. Finally, both lists were filtered according to variant coverage, and we kept only the variants having at least 1, 2, 3, 4, or at least 5, supporting reads. The numbers of variants returned by this procedure are listed in Table 1. It should be noticed that, due to the random nature of sequencing errors and the large coverage, there is a very large number of false positives. They also decrease more slowly than the true positives as a function of read coverage. This fact demonstrates that, while the strategy of filtering calls based on coverage might be useful to increase the specificity of the method, it also leads to the irrecoverable loss of a large number of likely real variants.

We also generated a control set of random variant calls as follows. If a base is not the consensus base according to the pileup at some position, we treated it as a potential variant at that position. From these potential variants, we selected random sets having different sizes. The sizes of the sets were chosen to range from the size of the smallest set of predictions the other methods had made up to the size of the largest, in increments of 500. As the potential variants were selected randomly irrespective of their coverage and quality, we would expect this method not to show any special sensitivity or specificity, which is exactly what we observed. Thus, this method can be treated as a control, and used to make sure that there are no major underlying mistakes in the evaluation procedure.

## 3. Results

There are numerous low-frequency variant callers, some of which are reviewed in the introduction. In this section, we show how SiNPle compares with two low-frequency callers, LoFreq and VarScan2. We chose them because they are widely cited and used for general purpose low-frequency variant calling. In addition, they represent the first step of several analysis worflows, for instance DiversiTools [21].

### 3.1. Assessing Specificity and Sensitivity of Variant Calling in Simulated Data

**Figure 2:**
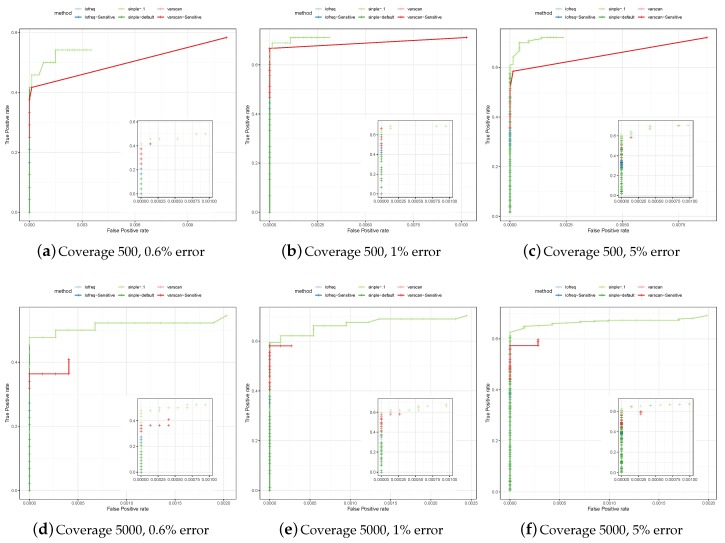
Comparison of variants discovered on simulated poliovirus data by SiNPle (Simplified Inference of Novel Polymorphisms from Large coveragE), LoFreq and VarScan2. We simulated coverages of 500 and 5000, and for each coverage value three different levels of error: 0.6%, 1%, and 5%.

To test specificity and sensitivity of the considered variant calling methods in a controlled setup, we generated a set of reads simulated from a poliovirus (genome size 7.5 kbp) having different mutation rates and coverages (see Section 2). The set of true variants is known, and called variants that are not in that set are classified as false positives. Figure 2 shows the Receiver-Operator Curves (ROCs) on these simulated datasets.

We ran each of the methods considered—SiNPle, VarScan2 and LoFreq—both with its default parameters and in a more sensitive mode, which depends on the method and is described in Section 2. The ROCs show that LoFreq and VarScan2 tend to be very conservative when calling variants in their default modes. While they exhibit a high specificity, the sensitivity is very low. The results of LoFreq in its sensitive mode are similar to those in the default mode. VarScan2 does capture many more variants in its sensitive mode, but the specificity starts to get affected rapidly. SiNPle shows a consistently higher sensitivity than that of all other callers (in particular VarScan2 in sensitive mode), even at its most stringent settings, and a similar specificity. In general, SiNPle strikes a very good balance between sensitivity and specificity if compared to other methods.

### 3.2. Comparative Assessment of Poliovirus Predictions

**Figure 3:**
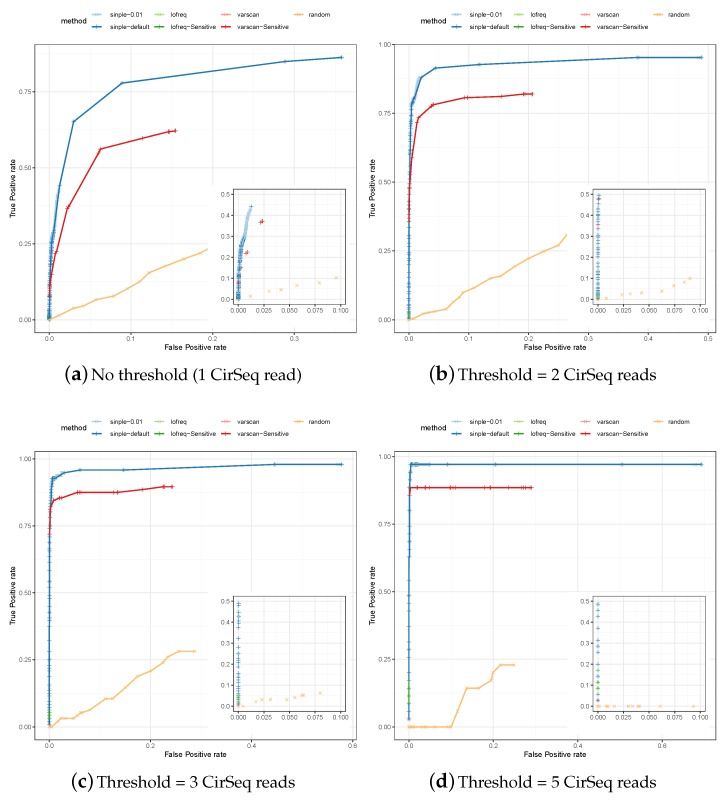
The Receiver-Operator Curves (ROCs) for the specificity and the sensitivity of variants predicted by SiNPle, LoFreq, VarScan2 (both standard and sensitive mode), and a control method based on the random selection of variable positions on the genome irrespective of whether they are true variants or not (see Section 2), from an input of 100,000 reads randomly extracted from a poliovirus dataset sequenced with CirSeq [19]. CirSeq allows the experimental discovery and validation of very low-frequency variants present at population level. The figures were obtained by setting different thresholds on the lists of true and false positives identified by calling consensus on CirSeq reads (see Section 2). All the three methods were run in both a default and a sensitive mode (see Section 2 for their definitions).

We also used data generated with the CirSeq protocol [19] to identify a set of true and false positives. The ROCs for the predictions of SiNPle, LoFreq, VarScan2 (each method run both in its default and in a more sensitive mode), and the random method described in Section 2, are shown in Figure 3.

The results are fully consistent with those for simulated data, with: the default modes of LoFreq and VarScan2 showing high specificity by very low sensitivity; the sensitive mode of LoFreq giving similar results; and the sensitive mode of VarScan2 showing better sensitivity but being consistently worse than SiNPle. In particular, SiNPle achieves excellent results for this dataset—with a threshold of two reads or higher, the optimal points on the ROC curve for SiNPle have a false positive rate <0.05 and a true positive rate >0.9. SiNPle is able to discriminate real variants well even at threshold 1, i.e. when all variants are considered and coverage can be very low. Thus, the ROC curves for SiNPle show an excellent trade off between sensitivity and specificity compared to other programs. As is to be expected, the fractional sensitivity of all callers increases with the coverage threshold, but the absolute number of caller variants decreases dramatically—i.e., most of the low-coverage variants are missed by all considered methods apart from SiNPle.

In Table 2, we also show in detail the predictions formulated by all the methods in the set of true positive variants when a coverage threshold of five CirSeq reads is considered (the lists generated for lower thresholds would be too long). In general, the sensitivity of LoFreq and VarScan2 drops with the frequency of the variant, although of course other factors (such as the total number of reads at that position, quality values, etc.) will also play a role. Consistent with the ROC curves shown above, SiNPle in its default setting was able to detect the largest number of variants—and the proportion of variants missed by the other methods would be even larger at lower thresholds.

### 3.3. Variant Calling on Different Viruses

We also ran a comparison on other real-life Illumina datasets. As we do not know the ground truth for them, we assumed that the conclusions drawn in the previous sections can be generalised to other datasets; hence, we set out to measure the number of variants found by each program as a rough proxy for their sensitivity. The viruses considered were IBV (genome size 27 kbp) and HIV (genome size 9 kbp). The viruses were assembled with an in-house pipeline using SPAdes [22] and additional custom software; the resulting assembly was used as the reference genome. Datasets were then purified using flexbar (Version 3.4.0) [23] and mapped using the GEM mapper [24] (version 3) with default settings to the reference genomes. The alignments were processed with samtools (Version 1.9) to create sorted BAM files and their pileups. Pileups were generated with coverage set to a very large number to ensure that no pileup information was lost. LoFreq was run on the final BAM files and the other variant callers were run on the pileups. All software was run with default settings on a single Intel^®^ Xeon^®^ E5-2620 v3 CPU core at 2.40 GHz (computer cluster assembled by Bull SAS, Paris, France). We chose SiNPle predictions with posterior probability ≥0.95.

**Figure 4:**
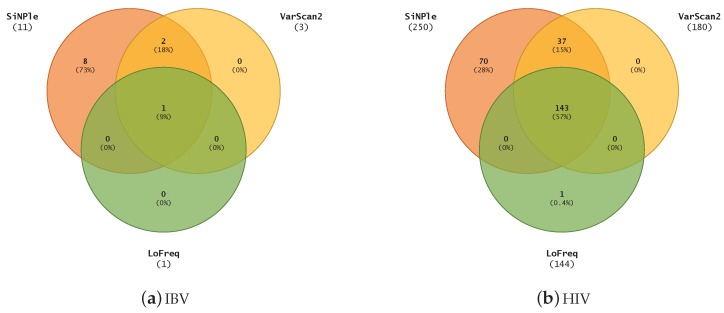
The comparison of variants discovered on IBV and HIV datasets using SiNPle, LoFreq and VarScan2 in their default modes.

Figure 4 shows the comparison of the results for all the methods considered. For IBV, SiNPle produces a superset of the other results. SiNPle discovered eight more SNVs than the others and one common variant was discovered by all three methods. For HIV, SiNPle results are virtually a superset of the others at default parameters, with only an extra prediction being made by LoFreq. However, SiNPle makes 70 additional predictions.

### 3.4. Speed Benchmarks

We also benchmarked the speed at which each aligner calls variants. While LoFreq accepts a BAM file as input, the others programs require a pileup as generated, for instance, by samtools mpileup. We separately computed the time taken to process the pileup for SiNPle and VarScan2, and added it to the runtime for a fairer comparison with LoFreq. One should also note that, albeit SiNPle and VarScan2 use external tools to compute the pileup, they still need to parse the result, which can be bulky. Table 3 shows the final results. In general, SiNPle is consistently faster than the other callers. This speedup becomes more apparent on the larger IBV dataset, on which SiNPle was almost 10× faster than LoFreq—in general, LoFreq seems to scale unfavourably when coverage increases. It should be noted that, although perhaps higher than the typical figures one would expect for lower-coverage datasets, the timings reported are quite typical.

## 4. Discussion

Computational discovery of variants from high throughput data is a non-trivial task. The core problem is in weeding out sequencing artefacts while retaining a high sensitivity to true variants. We propose a fast Bayesian statistical framework that can generate posterior probabilities for a variant to be true. Our benchmarks show that our method runs faster than other popular variant callers, and in some cases the speed advantage can be close to an order of magnitude. By changing the prior probability values, SiNPle users can find a suitable trade-off between sensitivity and specificity of their variant calling.

The major parameters that SiNPle requires are priors about sequencing and other errors—for instance, the prior pairwise nucleotide diversity per base in the viral population. Such values cannot be exactly known in advance. However, we can assign suitable values based on prior knowledge of similar populations. In Section 3, we show that it is easily possible to change parameters in order to make SiNPle attain the best specificity/sensitivity trade-off.

There are several commonly used pipelines that are based on LoFreq and VarScan2. For example, DiversiTools [21] usually relies upon LoFreq to identify low-frequency variants. Both our real-life and simulated data experiments showed that such a choice is a poor one, because LoFreq consistently favours specificity over sensitivity. For such cases, a switch to SiNPLe would be extremely beneficial, giving easy access to a fuller spectrum of variants.

SiNPle scales well with the size of the datasets. The main time spent is in externally parsing the pileup. A good rule of thumb to get an upper bound for the timings in the case of larger genomes with the same coverage would be to divide the timings we report by the length of the virus, and multiply them by the length of the desired genome. If the coverage is lower than the ones we report in our experiments by some factor, one would gain an additional speed-up of at least that factor—the computation of the pileup scales at least linearly with the number of reads.

Overall, we are convinced that the advantage of speed, the possibility of fine-tuning and the excellent specificity and sensitivity will make SiNPle a valuable tool while performing variant discovery out of deep sequencing data.

## Figures and Tables

**Table 1:** Number of false and true positive variants returned by the selection procedure of Figure 1 as a function of the coverage threshold.

Coverage Threshold	1	2	3	4	5
True positives	1125	233	96	59	35
False positives	10,158	4739	2386	1302	739

**Table 2:** Variants called by SiNPle, Lofreq and VarScan2 on 100,000 randomly sampled CirSeq reads. The table also shows the number of consensus reads from CirSeq that validate the second most frequent genotype. The variants in the table are the list of true positives thresholded at coverage 5 (see Section 2) and hence they are all likely to be real. We chose coverage 5 in order to obtain a table of reasonable size; other values produce similar results (see Figure 3).

Position	Genotype	Supporting Reads	LoFreq Default	Sensitive	SiNPle Default	VarScan2 Default	Sensitive
2133	C	172	✓	✓	✓	✓	✓
4348	A	42	✓	✓	✓		✓
6952	T	15			✓		✓
2456	C	13			✓		✓
4357	C	13			✓		✓
1867	G	11			✓		✓
2547	A	10	✓	✓	✓		✓
4104	C	10	✓	✓	✓		✓
1870	A	8			✓		✓
3255	G	8		✓	✓		✓
4994	G	8			✓		✓
5091	A	8			✓		
5233	C	8			✓		✓
6937	C	8			✓		✓
2009	T	7			✓		✓
3950	T	7			✓		✓
4100	A	7	✓	✓	✓		✓
6224	T	7			✓		✓
3978	A	6			✓		✓
4203	G	6			✓		✓
5143	T	6			✓		✓
5192	A	6			✓		✓
6802	G	6			✓		✓
7029	T	6			✓		✓
2088	T	5			✓		✓
2684	T	5			✓		✓
3690	C	5			✓		✓
4356	C	5			✓		
6234	T	5			✓		✓
6413	T	5			✓		✓
6477	A	5			✓		
6508	G	5			✓		✓
7080	G	5			✓		✓
7320	G	5			✓		✓

**Table 3:** Time taken in seconds to process the IBV, HIV and poliovirus datasets on a single core using default settings. The time is the average of three runs, and figures for SiNPle and VarScan2 include the time spent on generating the pileup with samtools pileup. For LoFreq, pileup generation is not a factor as LoFreq works directly with BAM files.

Dataset	Reads	Pileup	SiNPle	VarScan2	LoFreq
HIV	74,996	20.5	23.4	43.2	72.3
Poliovirus	100,000	34.8	36.6	45.7	51.2
IBV	3,778,012	690	755	910	7,324

## Abbreviations

The following abbreviations are used in this manuscript:

SNP	Single nucleotide polymorphism
NCBI	National Center for Biotechnology Information
SRA	Short Read Archive
ROC	Receiver-operator curve
IBV	Infectious bronchitis virus
HIV	Human immunodeficiency virus.
